# Supplementary material for: ZnT8 Loss of Function Mutation Increases Resistance of Human Embryonic Stem Cell-Derived Beta Cells to Apoptosis in Low Zinc Condition
Source: Cells. 2023 Mar 15;12(6):903. doi: 10.3390/cells12060903 (PMC10047077; doi:10.3390/cells12060903)
Supplement: Supplementary file 1 [file cells-12-00903-s001.zip › cells-2154423-supplementary_02242023.pdf]

Supplementary Figure 1.

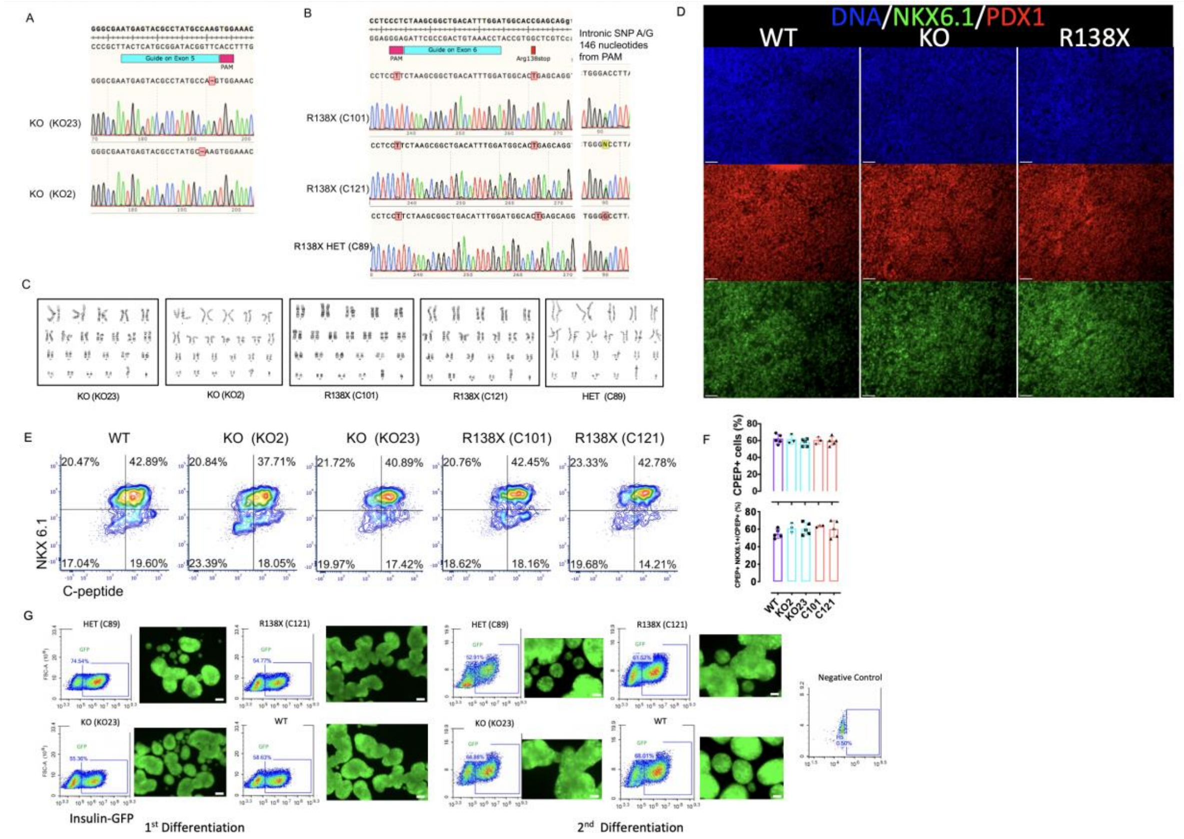

Supplementary Figure S1. Establishment of *SLC30A8* mutants with CRISPR/Cas9 and their differentiation towards insulin-producing cells. (**A, B**) Sanger sequencing chromatograms show the position of introduced mutation in (A) two clones of KO cell lines (KO23 and KO2), (**B**) two clones of R138X homozygous cell lines (C101 and C121), one clone of R138X heterozygous cell line (C89). (**C**) Karyotypes of cell clone KO23, KO2, C121, C101 and C89. (**D**) Representative immunostaining showing separated channel for NKX6.1, PDX1 and DNA in WT, KO and R138X for **Fig. 1B**. (**E**)Representative FACS analysis of NKX6.1 and C-peptide-positive cells in WT, KO and R138X. (**F**) Quantification of C-peptide-positive cells (%) and C-peptide and NKX6.1 double-positive out of C-peptide positive cells (%) in each clone of WT, KO and R138X cell lines. (**G**) Flow cytometry quantification of insulin-GFP-positive cells derived from R138X heterozygous (C89), R138X homozygous (C121) and KO (KO23). WT stem cells served as negative control. (All analyses were done on day 27 of differentiation. Scale bar: 100 mm. Related to Figure 1.)

Supplementary Figure 2.

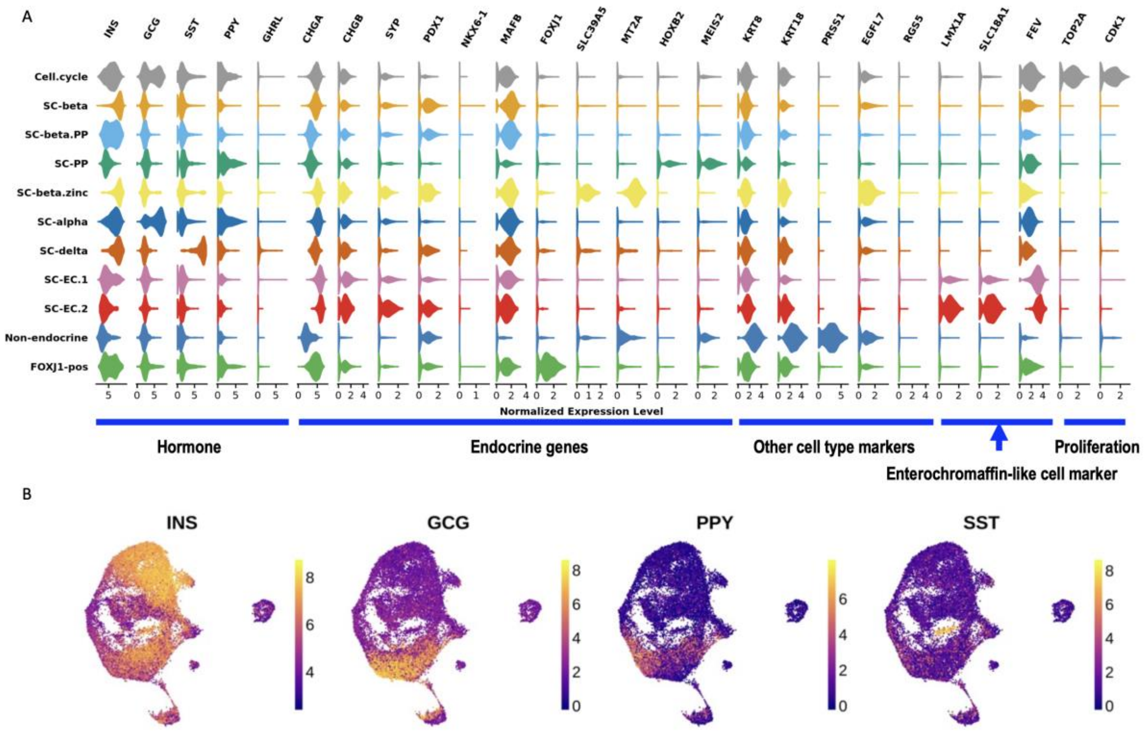

Supplementary Figure S**2. Single cell RNA sequencing analysis in representative sc-islet like clusters.** (**A**) Single cell RNA sequencing identified 10 endocrine and 1 non-endocrine subpopulations in sc-islet like clusters. (**B**) The expression levels of selected endocrine markers INS, GCG, PPY and SST in each identified population by single cell RNA sequencing.

Supplementary Figure 3.

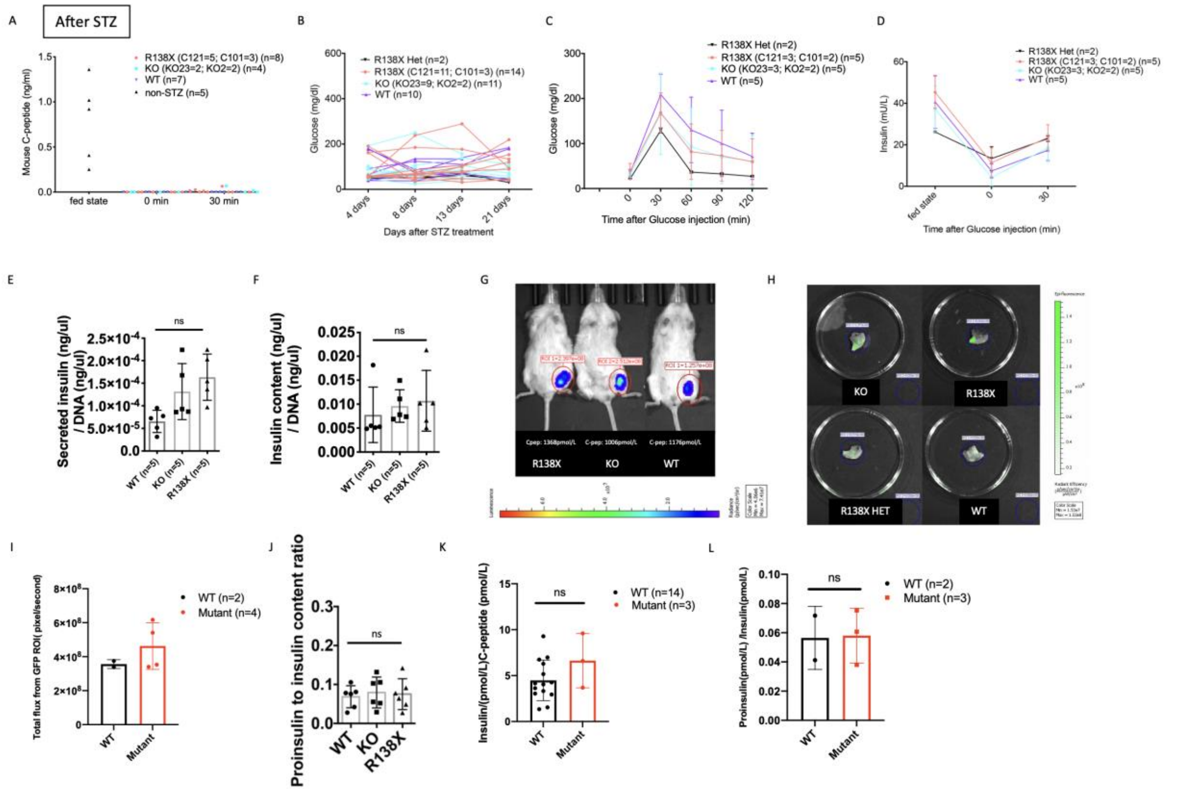

Supplementary Figure S**3. Comparable functions of sc-beta cells derived from S*LC30A8* KO, R138X heterozygous and homozygous mutant cells and WT cells after transplantation.** (**A**) Mouse C-peptide secretions were measured in WT (n=7), KO (n=4), R138X (n=8) at fasting and 30 min of glucose injection after STZ mediated ablation of mouse beta cells. Non-STZ mice were served as controls for mouse C-peptide secretion measured at fed state (n=5). (**B**) Blood glucose levels of STZ-treated mice transplanted with each indicated genotype of each cell line. (**C**) Glucose tolerance test on STZ-treated mice with normal levels of blood glucose in fed state, fasting state, 30, 60, 90 and120 min after glucose injection. (**D**) Human insulin secretion in STZ-treated mice transplanted with cell lines with each indicated genotype in fed state, fasting state and 30min after glucose injection. (**E**) Representative bioluminescence intensity (pixel/second) of grafted cells in mice transplanted with WT, KO and R138X cells. Human C-peptide level for the mice presented are respectively: 1368, 1006 and 1176 pmol/L. (**F**) Representative GFP intensity in isolated grafts derived from mice transplanted with WT, KO, R138X homozygous and heterozygous

sc-beta cells. (**G**) Quantification of beta-like cells GFP intensity in (F). Grafts analysis *in vivo* in (E, F, G) were done at 13 months after transplantation. (**H**) Ratio of insulin content to C-peptide content in WT and SLC30A8 mutant grafts. Noted that mice transplanted with WT sc-beta cells includes data from (Gonzalez et al., 2022). Statistical analysis using One-way ANOVA, ns: not significant. (**I**) Ratio of proinsulin content to insulin content in grafts of WT and SLC30A8 mutant (including KO and R138X homozygous mutation). (**J**) Basal insulin secretion of WT, KO and R138X sc-beta-like cells at 30 min with 2mM glucose solution after pre-incubation with 2mM glucose for 1h. One-way ANOVA with *$p<0.05$. (**K**) Insulin content of WT, KO and R138X stem cell-derived beta-like cells after normalization to the DNA content. One-way ANOVA with ns: not significant. (**L**) Ratio of proinsulin to insulin content of WT, KO and R138X sc-beta-like cells. One-way ANOVA with ns: not significant.

Supplementary Figure 4.

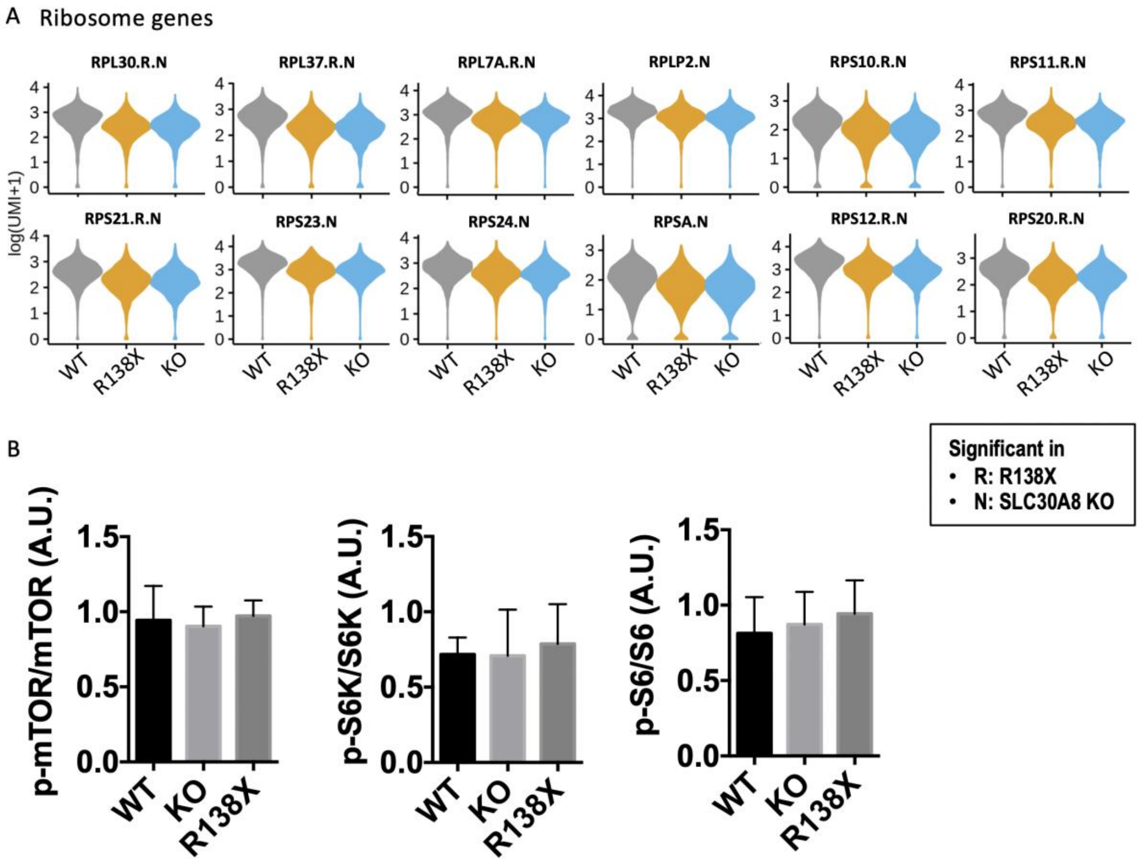

Supplementary Figure S4. Loss of SLC30A8 expression does not alter the mTOR signaling activity. (**A**) The expression of ribosome genes in WT, R138X and KO mutant sc-beta cells. (**B**) Quantification of western blot band intensity of p-mTOR, p-S6K and p-S6 and normalized to each total form of protein (n=3).

Supplementary Table S1. Primary Antibodies List

Primary Antibody List				
Antibody	Species	Dilution	Company	Catalog No.
C-peptide	Rat	1:100	DSHB	GN-ID4
Glucagon (*in vitro*)	Guinea Pig	1:200	Takara	M182
Glucagon (*in vivo*)	Mouse	1:1500	Sigma-Aldrich	G2654
NKX6.1	Mouse	1:300	R&D System	F55A10
PDX1	Goat	1:100	DSHB	AF2419
ZNT8	Rabbit	1:100	Abcam	Ab254577

Supplementary Table S2. Secondary Antibodies List

Secondary Antibody List			
Antibody	Dilution	Company	Catalog No.
Donkey anti-rat IgG Alexa Fluor 488	1:500	Thermo Scientific	A-21208
Goat anti-guinea pig Alexa Fluor 488	1:500	Thermo Scientific	A-11073
Alexa Fluor$^{@}$ 555 Donkey anti-mouse IgG	1:500	Life Technologies	A31570
Alexa Fluor$^{@}$ 647 AffiniPure Donkey Anti-guinea pig IgG	1:500	Jackson ImmunoResearch Laboratories	706-605-148
Donkey anti-goat Alexa Fluor 555	1:500	Life Technologies	A-21432
DNA stain Hoechst 33342	1:500	Life Technologies	H3570

Supplementary Table S3. Guide sequence and repair template

	Guide sequences (***PAM***)	ssDNA template
SLC30A8 KO (KO2/KO23)	ATGAGTACGCCTATGCCAAG***TGG***	N/A
SLC30A8 R138X (C101/C121)	TCCAAATGTCAGCCGCTTAG***AGG***	CTCTTCTCCCTGTGGTTGTCATCGAAGCCTCCATCTAAGCGGCTGACATTTGGATGGCACTGAGCAGGTACGGTTCATAGAGTGAGCAATAACAGCAGGCTGGTGCTGCAAAGTCAAACC

Supplementary Table S4. Differentially expressed genes in sc-beta cells among WT, KO and R138X. (See excel file: Supplementary Table 4)
